# The incidence of invasive pneumococcal serotype 3 disease in the Danish population is not reduced by PCV-13 vaccination

**DOI:** 10.1016/j.heliyon.2016.e00198

**Published:** 2016-11-29

**Authors:** Hans-Christian Slotved, Tine Dalby, Zitta Barrella Harboe, Palle Valentiner-Branth, Victoria Fernandez de Casadevante, Laura Espenhain, Kurt Fuursted, Helle Bossen Konradsen

**Affiliations:** a Neisseria and Streptococcus Reference Laboratory, Department of Microbiology and Infection Control, Statens Serum Institut, Copenhagen, Denmark; b Department of Infectious Diseases, Rigshospitalet, Copenhagen University Hospital, Copenhagen, Denmark; c Department of Infectious Disease Epidemiology, Statens Serum Institut, Copenhagen, Denmark

**Keywords:** Medicine, Immunology, Infectious diseases

## Abstract

Since 2010, Denmark has included the 13-valent pneumococcal conjugated vaccine (PCV-13) in the childhood immunization programme. However, serotype 3 remains as an important cause of invasive pneumococcal disease (IPD) in Denmark. IPD surveillance data (1999–2016) was used to calculate the incidence and age-distribution of serotype 3 IPD, and the effect of PCV-13 on serotype 3 IPD incidence was examined. The incidence of serotype 3 IPD in the age group below 65 years was 0.51/100,000 pre PCV-13, and 0.45/100,000 post PCV-13. In the group 0–4 years, serotype 3 IPD incidence was 0.28/100,000 pre PCV-13, and 0.16/100,000 post PCV-13. Serotype 3 IPD incidence in the elderly showed a mean of 4.27/100,000 pre PCV-13, and 4.32/100,000 post PCV-13. PCV-13 childhood immunization in Denmark has not lead to a reduction of the incidence of IPD caused by serotype 3. The reason behind this missing effect needs to be investigated further.

## 1 Introduction

*Streptococcus pneumoniae* (pneumococcus) causes non-invasive as well as invasive infections, and invasive pneumococcal disease (IPD) is a cause of high morbidity and mortality particularly among young children and the elderly [1]. The introduction of the pneumococcal conjugate vaccines (PCV) for infants has lead to a marked decrease in IPD among young children and has also lead to a decrease in IPD among the general population through a herd protection effect. This effect has been seen in many countries around the world [2, 3, 4, 5].

In Denmark, a 2 + 1 vaccination schedule of the PCV7 (Prevenar 7, Pfizer Vaccines) was introduced in 2007 (3, 5 and 12 months of age) and was replaced by the PCV13 (Prevenar 13, Pfizer Vaccines) with the same schedule in 2010 [2]. The uptake of the PCVs among Danish children has been stable and for the coverage is around 80% for the final (third vaccination) PCV vaccination at 12 months of age, [www.ssi.dk/data]. The general effect of the PCV vaccines in Denmark has previously been described [2].

Although most cases of IPD caused by serotypes included in the vaccines has decreased markedly in the Danish population, this is not the case for serotype 3. The incidence of serotype 3-IPD has not changed significantly in the overall population after the introduction of PCV13 vaccination, other than what would be expected from natural cyclical patterns [2]. Although PCV vaccine failures in children have been reported, they are considered to be rare and mostly found in combination with some degree of immunosuppression [6, 7]. Vaccine failure are therefore not the reason for the serotype 3 observation.

With this study, we present national surveillance data on serotype 3- IPD from a 17-year period in order to further explore the burden of serotype 3 disease in the Danish population and thereby seek an explanation to why a reduction in serotype 3 IPD has not been observed.

## 2 Materials and methods

The description of the study population and laboratory identification of pneumococcal isolates is identical to the description made in the study by Slotved et al. [8]. Parts of the surveillance data have previously been presented [2, 8], except that surveillance data for serotype 3 in the period of 2015 and 2016 has been added.

## 3 Study population

Data on serotype 3 invasive pneumococcal isolates from January 1999 to June 2016 was retrieved from the Danish laboratory surveillance system at the National Neisseria and Streptococcus Reference Laboratory (NSR), Statens Serum Institut (SSI). The surveillance system only describes basic epidemiology regarding IPD cases with limited information on known risk factors for the individual cases, however if there are suspected cases of vaccine failures in children, detailed information on possible risk factors can be collected. Information regarding the surveillance system and risk factors can be found in Harboe et al. [6].

A case of IPD was defined as the isolation of *S. pneumoniae* in cerebrospinal fluid, blood or other normally sterile sites [2].

Information on age, sex, sample collection date, serotype and clinical foci (blood, cerebrospinal fluid etc.) is included in this database. The coverage of the database has been described in detail previously [2]. Since October 2007, it has been mandatory for all clinical microbiology laboratories in Denmark to submit all isolates causing IPD to SSI for serotype identification and registration [9].

Population data were obtained from Statistic Denmark (www.dst.dk).

## 4 Laboratory identification of pneumococcal isolates

The pneumococci were identified by optochin susceptibility and bile solubility tests. All isolates were serotyped either by Quellung reaction alone or by the Pneumotest Latex kit (SSI-Diagnostica, Copenhagen, Denmark) combined with the Quellung reaction using type-specific pneumococcal rabbit-antisera as previously described (SSI-Diagnostica, Copenhagen, Denmark) [10, 11].

## 5 Data analysis

Data were analysed using Graph Pad Prism version 5 (GraphPad Software) for descriptive statistical analysis. All calculations of incidence rate, incidence rate ratio (IRR) and confidence interval (CI) were performed using R version 3.2.4 Revised (2016-03-16 r70336) Copyright (C) 2016 The R Foundation for Statistical Computing Platform: x86_64-w64-mingw32/x64 (64-bit) (http://www.r-project.org/). Lower CI values calculated to be below 0 were set to 0.

Fisher's Exact Test in R was used to calculate P-values. P < 0.05 was considered significant. Serotype 3-IPD cases are presented stratified by age groups.

The total number of serotype 3 IPD cases for the year 2016 was estimated based on the number of cases during the first six months and then adjusted for seasonal variation by the use of IPD data from 1999 to 2015. These data showed that 60.6% of all cases on average occurs during the first six months of the year.

## 6 Ethical considerations

The study was a retrospective, population-based study based on national laboratory surveillance data on isolates from patients with IPD. Since data and samples from patients were collected routinely for national surveillance purposes, no ethical approval or informed consent from patients or guardians were required. The study was approved by the Danish Data Protection Agency (record number 2007-41-0229).

## 7 Results

**Fig. 1:**
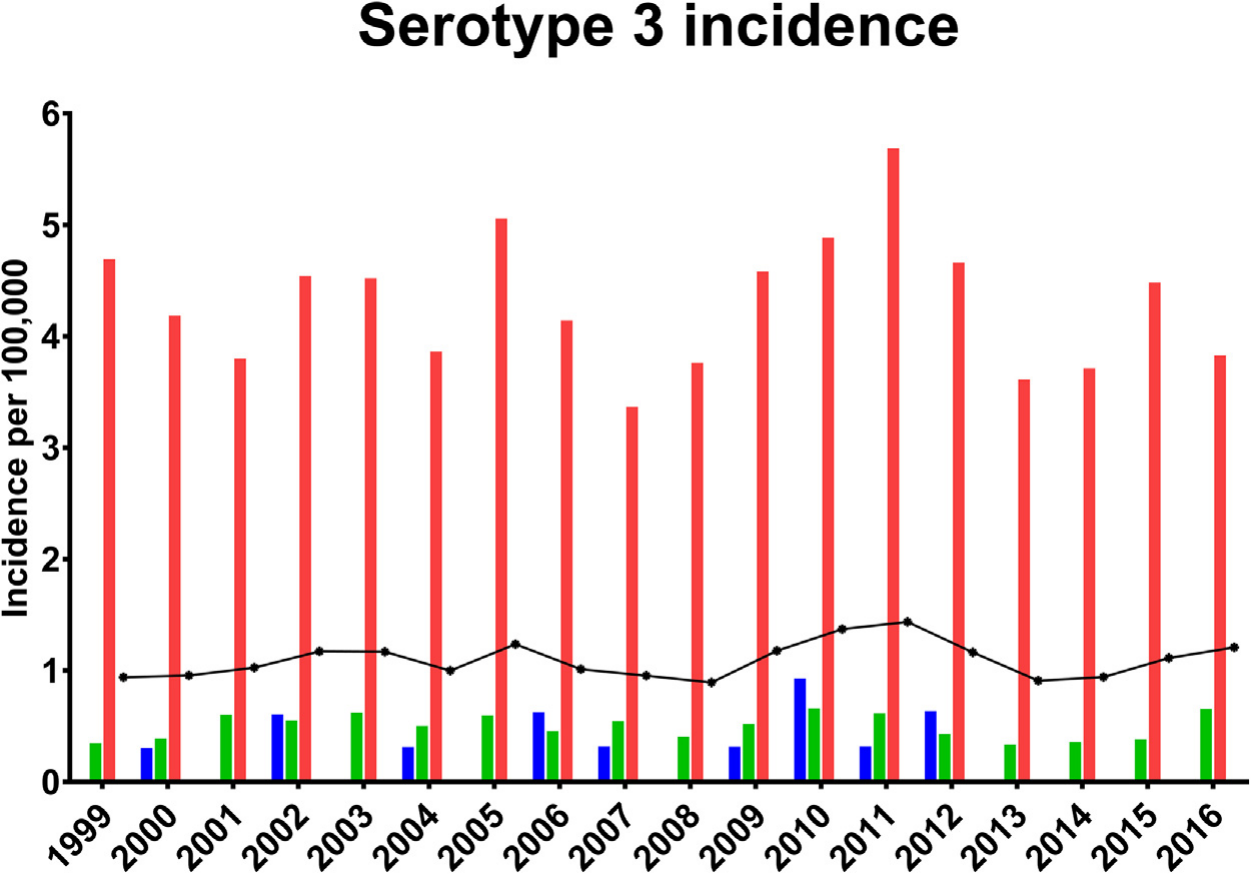
The IPD incidence with the corresponding 95% CI indicated as error bars for serotype 3 in all three age groups from 1999 until 2016. Blue bars represent 0–4 years, green bars represent 5–64 years and red bars represent 65+ years. The curve represents the total incidence per year. The 2016 data is corrected for seasonal variation (IPD/60.6)*100.

Detailed data of the incidence of serotype 3 IPD cases in three age groups from 1999 until 2016 are presented in Fig. 1 and Table 1. In general, serotype 3 IPD cases in the age group 0–4 years were rare prior to the introduction of PCV-7 in 2010, with an incidence of 0.28 per 100,000 (95%, Confidence Interval [CI] 0.09–0.47) and an incidence of 0.16 per 100,000 (95%, CI. 0–0.43) in 2010 when PCV-13 was introduced (Table 1). No major differences in the IRR after introduction of the vaccines was seen. This was also observed for the age group 5–64 years, showing very low incidences of serotype 3 IPD cases both before (0.51 per 100,000, 95%, CI. 0.44–0.57) and after (0.45 per 100,000, 95%, CI. 0.31–0.60) PCV-13 introduction.

The incidence of serotype 3-IPD in the age group +65 was 4.27 per 100,000 (95%, CI. 3.95–4.60) prior to PCV-13 introduction, and 4.32 per 100,000 (95%, CI. 3.49–5.15) after the introduction PCV-13 (Table 1). There was no statistical significant difference between the incidences for the two periods.

## 8 Discussion

This study indicates that PCV vaccination of children in Denmark has not induced a herd protection for serotype 3 IPD cases in the elderly. An indication of this situation was observed in the study by Harboe et al. [2] and Slotved et al. [8], where serotype 3 IPD data from 1999 to 2014 were presented. The Mandatory laboratory notification of IPD cases was started at the same time as PCV-7 was introduced in 2007, which means that there has been three years of prior experience of notification before the introduction of PCV-13 was included. The PCV-13 in general is not used in the elderly in Denmark, and there is good knowledge of PCV vaccine failures in children [6]. According to Harboe et al. [12], SSI received between 96% and 100% of all *S. pneumoniae* isolates obtained from cerebrospinal or blood cultures in 2004 and 2005. Based on that, the notification of IPD in Denmark is considered to be trustworthy also before 2007, where the notification was made mandatory. Issues with the notification system is therefore not considered to be a factor associated with the epidemiology of serotype 3 IPD in Denmark.

This study furthermore shows that serotype 3 has not been a problem in children since 1999, with only 14 reported IPD cases from January 1999–June 2016 (Fig. 1). Pneumococcal carriage, IPD incidence, and herd protection effect for the unvaccinated age groups in relation to the PCV vaccines in Denmark have previously been described and overall a good effect of the vaccines has been shown [2, 8]. The focus in recent years has therefore turned to predicting which non-vaccine IPD serotypes will emerge (serotype replacement) and several studies from around the world have shown data on the most common non-PCV serotypes in their region [3, 8]. However, as has also been observed in several studies [13], PCV-13 has not had an equal effect for the thirteen serotypes covered by the vaccine, and in Denmark the current situation is that serotype 3 is still a common cause of IPD in the elderly. On the recent pneumococcal meeting (ISPPD-10) in Glasgow, several countries reported a similar situation, with increasing IPD incidences caused by some of the PCV-13 serotypes in the non-vaccinated age groups, including serotypes 3 and 19A [14, 15, 16, 17]. Children are traditionally the primary target for conjugate pneumococcal vaccination because they have the highest incidence of disease but also carriage [3, 18]. PCV vaccination programmes for children have in general led to that the majority of the PCV serotypes have become rare in both children and adults due to a combination of immunity and herd protection [2, 3, 18]. It has also been shown that PCV reduces carriage [18]. The reasons behind the relatively high incidence of serotype 3 disease in adults are unknown. One hypothesis could be that serotype 3 might be carried predominantly in the adults in our population. Supporting this assumption is the fact that serotype 3 IPD cases have not been observed in children since 2012 (Fig. 1). However, there is no data available, including post PCV vaccination carriage data, that could provide evidence for any explanation of why the number of serotype 3 IPD cases are unaffected by the PCV-13 vaccination of children in Denmark.

Finding out the reasons why serotype 3 IPD cases have not been reduced in Denmark seems very important, since other serotypes show a similar IPD pattern as serotype 3 among the different age groups. Among these serotypes is serotype 8, a non-PCV vaccine serotype, which has been the most common cause of IPD in Denmark for the last couple of years [8]. However, this serotype is not a problem in children (age group 0–4 years) [8]. Serotype 8 IPD incidence might therefore be equally non-affected by a vaccine as the serotype 3 IPD incidence, if serotype 8 was to be included in a PCV childhood vaccination programme.

In conclusion, this study indicates that childhood immunization with PCV-13 in Denmark seems not to have had an impact on the overall serotype 3 IPD incidence. This is not linked to vaccine failures, but must be due to other reasons, which will need to be further investigated.

## Declarations

### Author contribution statement

Hans-Christian Slotved: Conceived and designed the experiments; Analyzed and interpreted the data; Wrote the paper.

Tine Dalby, Zitta Barrella Harboe, Palle Valentiner-Branth, Victoria Fernandez de Casadevante, Laura Espenhain, Kurt Fuursted, Helle Bossen Konradsen: Analyzed and interpreted the data; Wrote the paper.

### Funding statement

This research did not receive any specific grant from funding agencies in the public, commercial, or not-for-profit sectors.

### Competing interest statement

Hans-Christian Slotved is participating in a project supported by Pfizer. The other authors declare no conflict of interest.

### Additional information

No additional information is available for this paper.

## Figures and Tables

**Table 1:** For all three age groups, the mean incidence rates and 95% confidence intervals are presented for serotype 3. Incidence Rate Ratios (IRRs) are calculated by mean incidence from one period divided by mean incidence from another period. None of the IRR values were statistically significant (P < 0.05 calculated using Fisher's Exact Test).

Serotype 3	1999–2010 (Before introduction of PCV-13)	2011–2016 (After introduction of PCV13)	1999–2010 versus 2011–2016
Age group	Mean incidence per 100,000, 95% CI (lower CI; upper CI)		IRR, 95% CI (lower CI; upper CI)
0–4 years	0.28 (0.09–0.47)	0.16 (0–0.43)	0.58 (0.10–2.21)
			P = 0.5694
5–64 years	0.51 (0.44–0.57)	0.45 (0.31–0.60)	0.89 (0.71–1.11)
			P = 0.326
65+ years	4.27 (3.95–4.60)	4.32 (3.49–5.15)	1.00 (0.86–1.17)
			P = 0.9685
